# Validation of the American Association for the Study of Liver Disease/European Association for the Study of the Liver Multistep Screening Strategies for Metabolic Dysfunction-associated Steatotic Liver disease

**DOI:** 10.1016/j.gastha.2025.100747

**Published:** 2025-07-10

**Authors:** Clémence Marie Canivet, Marie Ongaro, Nicolas Conquet, Laurent Spahr, Nicolas Goossens

**Affiliations:** 1 Division of Gastroenterology and Hepatology, Geneva University Hospitals, Geneva, Switzerland; 2 Division of Transplantation, Geneva University Hospitals, Geneva, Switzerland

**Keywords:** Liver fibrosis, steatotic liver disease, MASLD, screening, transient elastography

## Abstract

**Background and Aims:**

Multistep algorithms for noninvasive identification of patients with metabolic dysfunction-associated steatotic liver disease (MASLD), such as the recent American Association for the Study of Liver Disease and European Association for the Study of the Liver MASLD guidelines aim to identify patients who require hepatology referral. We evaluated the performance of these algorithms in the National Health and Nutrition Examination Survey cohort.

**Methods:**

We analyzed 7768 adult participants from the National Health and Nutrition Examination Survey 2017–2020 with valid liver stiffness measurement (LSM) data. MASLD was defined as hepatic steatosis (controlled attenuation parameter measurement of ≥ 248 dB/m) with ≥1 cardiometabolic risk factor; significant fibrosis and advanced fibrosis were defined as LSM ≥8 kPa and ≥12 kPa respectively (with sensitivity analysis of 0–30kPa and using the FibroScan-aspartate aminotransferase or Agile algorithms).

**Results:**

A total of 44.8% (n = 3479) met metabolic risk criteria, 7.5% (n = 586) met raised Fibrosis-4 Index criteria, and 3.0% (n = 235) met referral criteria. The multistep pathway demonstrated high specificity but limited sensitivity for identifying MASLD with significant fibrosis (sensitivity 21%, 95% confidence interval [CI] 17%–25%) or advanced fibrosis (29%, 95% CI 23%–37%). Sensitivity was particularly low in key subgroups: age < 50 years (6%, 95% CI 3%–11%), women (15%, 95% CI 11%–21%) and patients without type 2 diabetes (16%, 95% CI 12%–20%). Sensitivity improved when targeting more advanced disease: 29% for advanced fibrosis (LSM ≥12 kPa), 54% for FibroScan-aspartate aminotransferase ≥0.67, 35% for Agile 3+ ≥0.68, and 66% for Agile 4 ≥ 0.57.

**Conclusion:**

A multistep screening strategy based on metabolic risk criteria, Fibrosis-4 Index, and LSM demonstrates high specificity but low sensitivity for detecting MASLD with significant fibrosis in a US-based populational cohort.

## Introduction

Metabolic dysfunction-associated steatotic liver disease (MASLD), previously known as nonalcoholic fatty liver disease (NAFLD), has emerged as the most prevalent chronic liver disease globally, posing a significant public health challenge. MASLD is defined as the presence of hepatic steatosis with the presence of at least 1 cardiometabolic risk factors and the absence of significant alcohol consumption.^1^, ^2^, ^3^ The disease spectrum encompasses a range of severities, from simple steatosis to metabolic dysfunction-associated steatohepatitis (MASH), fibrosis, cirrhosis, and ultimately, MASH-related hepatocellular carcinoma. Beyond liver-specific outcomes, MASLD is strongly associated with cardiovascular disease, chronic kidney disease, and malignancies, underscoring its systemic impact.^4^

Significant liver fibrosis is the strongest predictor of liver-related morbidity and mortality, making early detection essential for improved patient outcomes.^5^ However, the asymptomatic nature of MASLD in its early stages often complicates timely diagnosis, frequently leading to identification at an advanced stage when complications such as decompensated cirrhosis or hepatocellular carcinoma have already developed.

Early identification of individuals with MASLD and significant fibrosis is therefore crucial for timely intervention and prevention of adverse outcomes. Multiple society recommendations, including American Association for the Study of Liver Disease (AASLD), American Gastroenterological Association (AGA) and European Association for the Study of the Liver (EASL) recommend multistep strategies to identify patients with significant fibrosis referred to specialist hepatology care.^1^^,^^3^^,^^6^^,^^7^ AASLD and EASL have both released similar updated guidelines in 2024 advocating targeted case-finding in patients with steatosis on imaging, clinical risk of MASLD or presence of metabolic risk criteria (individuals with type 2 diabetes [T2D]), abdominal obesity with additional cardiometabolic risk factors, or persistently elevated liver enzymes). These stepwise diagnostic algorithms, incorporating sequential noninvasive tests such as Fibrosis-4 Index (FIB-4) and liver stiffness measurement (LSM) by transient elastography (TE), aim to triage patients for specialized hepatology care.^1^^,^^3^ However, the performance of the metabolic risk criteria suggested in the EASL guidelines, and the overall multistep pathways have not yet been validated in general populational cohorts. Furthermore, FIB-4 has shown suboptimal accuracy and sensitivity for liver fibrosis screening in large populational cohorts.^8^ Therefore, validation of multistep liver fibrosis screening algorithms in large, poplational cohorts is essential.

Using data from the National Health and Nutrition Examination Survey (NHANES) 2017–2020, this study aims to validate the diagnostic performance of the AASLD and EASL multistep screening strategies for identifying patients with MASLD and significant fibrosis needing specialized hepatology referrals in a representative US population.

## Patients and Methods

### Study Design and Population

We utilized data from the NHANES collected from 2017 to March 2020.^9^ NHANES is a nationally representative, ongoing research program designed to assess the health and nutritional status of adults and children in the United States. The survey combines interviews, physical examinations, and laboratory tests to collect comprehensive health data. Starting from the 2017–2018 cycle, NHANES began performing TE using FibroScan®, providing both LSM (fibrosis assessment) and controlled attenuation parameter (CAP) values (for steatosis assessment). Due to the COVID-19 pandemic, the datasets from the 2017-18 cycles were combined with the 2019–2020 cycles to form the NHANES 2017-March 2020 prepandemic dataset. Inclusion criteria were age 18 years or more with complete TE data. We excluded all subjects aged <18 years of age or those with ineligible or unavailable TE measurements (Table A1). Demographic and clinical data, including metabolic comorbidities, serum markers, and LSM/CAP readings, were analysed, stratified by metabolic risk population criteria (T2D, abdominal obesity with an additional metabolic risk factor, or elevated liver enzymes).

### Definitions and Criteria

MASLD was defined as the presence of hepatic steatosis accompanied by at least one cardiometabolic risk factor, in the absence of other causes of liver fat accumulation, such as significant alcohol consumption or other liver diseases.^1^^,^^2^ The 5 cardiometabolic risk factors included: overweight or obesity (body mass index [BMI] >25 kg/m^2^ or >23 kg/m^2^ in Asian populations); dysglycaemia or T2D (hemoglobin A1c ≥ 39 mmol/mol or fasting plasma glucose ≥ 5.6 mmol/L, or treatment for T2D); plasma triglycerides >1.7 mmol/L, high-density lipoprotein cholesterol ≤1.0 mmol/L in men and ≤1.3 mmol/L in women or lipid-lowering treatment; and blood pressure ≥130/85 mmHg or treatment for arterial hypertension.^1^ Metabolic dysfunction and alcohol-related liver disease (MetALD) was defined as the presence of hepatic steatosis and at least one cardiometabolic risk factor (as defined for MASLD) in individuals with alcohol intake between 20-50g/day for females and 30–60g/day for males.

Steatosis was defined by a CAP measurement of ≥ 248 dB/m ^1^^,^^10^ and significant liver fibrosis as LSM ≥8 kPa.

Based on the AASLD and EASL guidelines we implemented a multistage screening strategy. Of note the AASLD criteria for selecting the at-risk population are less clearly defined so we used the EASL metabolic risk criteria and although both guidelines suggest alternatives to TE as second line tests (including enhanced liver fibrosis [ELF]), we focused on TE as the most validated strategy and because other tests were not available. Based on the guidelines we defined the metabolic risk, raised FIB-4 and hepatology referral criteria as follows:^1^^,^^3^

1. *Metabolic risk criteria* were defined as presence of T2D, abdominal obesity with at least one additional cardiometabolic risk factor, or persistently elevated liver enzymes. As previous liver enzymes were not available, patients with AST or ALT ≥ 50IU/L at time of evaluation were included.
2. *Raised FIB-4 criteria* were defined as increased age-adjusted FIB-4 score ≥1.3 if age < 65 years or ≥2.0 if age was ≥ 65 years^11^ in patients who met the metabolic risk criteria.
3. *Hepatology referral criteria* were defined as subjects who met the metabolic risk, raised FIB-4 criteria and in addition had a FIB-4 score >2.67 or LSM ≥8 kPa.

**Figure 1:**
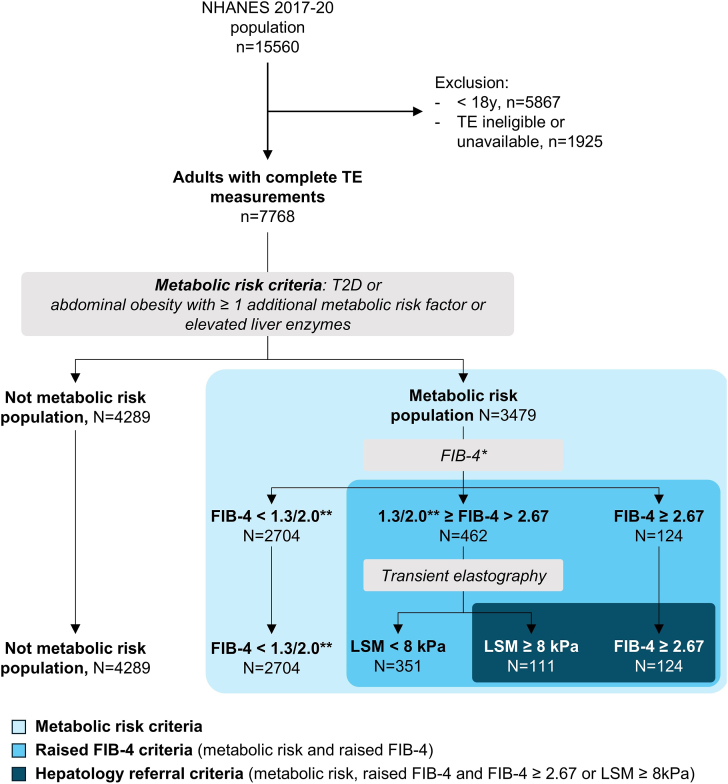
Flowchart of the study population selected from NHANES 2017–2020. After exclusion of individuals <18 years and those ineligible or without valid transient elastography (TE) measurements (n = 7792), the remaining 7768 participants with complete TE measurements were sequentially categorized using the metabolic risk criteria, raised FIB-4 criteria, and hepatology referral criteria. ∗n = 189 subjects lacked data for FIB-4 calculation. ∗∗ age-adjusted thresholds of FIB-4: >1.3 for individuals <65 years or >2.0 for those ≥65 years. FIB-4, Fibrosis-4 Index; LFTs, liver function tests; LSM, liver stiffness measurement; NHANES, National Health and Nutrition Examination Survey; TE, transient elastography; T2D, type 2 diabetes.

The *multistep screening strategy* refers to the overall referral strategy applying sequentially the above criteria (Figure 1). To examine the accuracy of the screening strategy we defined the primary target population for hepatology referral as MASLD or MetALD with significant fibrosis (MASLD with a LSM ≥8 kPa by TE^1^^,^^12^^,^^13^).

### Alternative Definitions of At-Risk Disease for Sensitivity Analyses

For sensitivity analyses, alternative definitions of at-risk individuals were explored using different LSM thresholds, specifically LSM ≥12 kPa and continuous LSM variations from 0 to 30 kPa. Additionally, previously published validated scores were used: a FibroScan-aspartate aminotransferase (FAST) score ≥0.67 was used to identify individuals at high risk of progressive MASH,^14^ an Agile 3+ score ≥0.68 helped identify individuals with MASLD and advanced fibrosis (≥F3),^15^ and an Agile 4 score ≥0.57 was employed for individuals with MASLD and cirrhosis (F4).^15^ These thresholds were selected as they were established in their respective original validation studies to provide specificity ≥90% for identifying target patients in their original publications.

### Statistical Analysis

Descriptive statistics were presented as median with interquartile ranges for continuous variables and number (%) for categorical data. Comparisons were conducted using the Wilcoxon rank-sum test for continuous variables and Fisher’s exact test for categorical variables. The diagnostic performance of each step of the *multistep screening strategy* was evaluated through sensitivity and specificity calculations, 95% confidence intervals (CIs) were calculated using Wilson score CI. Subgroup analyses examined diagnostic performance by age, BMI, and T2D status. All statistical analyses were performed using R (version 4.3.0).

### Modeling Analysis of the Performance of an Alternative Test to FIB-4

A modelling study was conducted to evaluate the diagnostic performance of a theoretical alternative test ("test B") replacing the FIB-4 step in the *multistep screening strategy*. The analysis focused on patients with metabolic risk criteria, simulating the diagnostic pathway where test B follows a positive metabolic risk screen. Test B sensitivity and specificity were systematically varied to assess their impact on combined sensitivity, specificity, and the Youden Index for identifying MASLD with significant fibrosis (LSM ≥ 8 kPa). Theoretical combined sensitivity and specificity were derived using established sequential testing formulas.^16^ Empirical combined performance was further validated through simulations with NHANES data. The Youden Index was used as an overall measure of diagnostic effectiveness and was calculated as sensitivity + specificity −1.^17^

### Ethical Approval

The National Center for Health Statistics Ethics Review Board approved the NHANES protocol (Protocol #2018-01) for the 2017–2020 cycle, and all participants provided written informed consent.

## Results

### Population Characteristics

A total of 15,560 subjects were initially identified from the NHANES 2017–2020 dataset. After excluding participants under 18 years (n = 5867) and those with ineligible or unavailable TE data (n = 1,925, detailed in Table A1), 7768 adults comprised our final study population (Figure 1). 45% of the cohort fulfilled the definition of MASLD (including 14% with significant fibrosis) and 7% had MetALD (including 11% with significant fibrosis) (Table 1 and Table A2).

**Table 1:** Baseline Characteristics of the Adult NHANES 2017–2020 Cohort With Valid Transient Elastography (TE) Data, Separated by Metabolic Risk Population

	All	Not metabolic risk population	Metabolic risk population	*P*-value
Demographic variables				
n (%)	7768	4289	3479	
Age (y): median (IQR)	50 (33–63)	44 (29–61)	55 (40–66)	<.001
Age ≥ 50 y (%)	3941 (50.7%)	1823 (42.5%)	2118 (60.9%)	<.001
Gender: male (%)	3862 (49.7%)	2120 (49.4%)	1742 (50.1%)	.584
Metabolic comorbidities				
Body mass index (BMI, kg/m^2^): median (IQR)	28.4 (24.5–33.3)	25.5 (22.8–28.2)	33.2 (30.2–37.4)	<.001
Weight (kg): median (IQR)	79.2 (67–94.5)	70.8 (61.2–80.7)	92.8 (80.2–107.1)	<.001
Arterial hypertension (%)	2820 (36.3%)	966 (22.5%)	1854 (53.4%)	<.001
Hyperlipidemia (%)	2673 (34.6%)	1090 (25.5%)	1583 (45.9%)	<.001
History of coronary heart disease (%)	292 (4%)	104 (2.6%)	188 (5.5%)	<.001
History of stroke (%)	342 (4.6%)	134 (3.4%)	208 (6.1%)	<.001
Type 2 diabetes (%)	1082 (13.9%)	0 (0%)	1082 (31.1%)	<.001
Serum variables				
Aspartate aminotransferase (U/L): median (IQR)	19 (16–24)	19 (16–23)	19 (16–26)	<.001
Alanine aminotransferase (U/L): median (IQR)	18 (13–26)	16 (12–22)	20 (14–31)	<.001
Gamma-glutamyl transferase (U/L): median (IQR)	21 (14–32)	18 (13–26)	25 (17–40)	<.001
Total bilirubin (μmol/L): median (IQR)	6.8 (5.1–10.3)	6.8 (5.1–10.3)	6.8 (5.1–8.6)	<.001
Serum albumin (g/L): median (IQR)	41 (39–43)	41 (39–44)	40 (38–42)	<.001
Serum creatinine (μmol/L): median (IQR)	74.3 (62.8–88.4)	73.4 (62.8–86.6)	75.1 (62.8–90.2)	<.001
Fasting glucose (mmol/L): median (IQR)	5.2 (4.8–5.7)	5 (4.7–5.3)	5.5 (5–6.5)	<.001
Glycated hemoglobin (%): median (IQR)	5.5 (5.2–5.9)	5.4 (5.1–5.6)	5.8 (5.4–6.6)	<.001
Triglycerides (mmol/L): median (IQR)	1.3 (0.9–1.8)	1.1 (0.8–1.5)	1.5 (1.1–2.2)	<.001
High-density lipoprotein (mmol/L): median (IQR)	1.3 (1.1–1.6)	1.4 (1.2–1.7)	1.2 (1–1.4)	<.001
Total cholesterol (mmol/L): median (IQR)	4.7 (4.1–5.4)	4.7 (4.1–5.4)	4.7 (4–5.4)	.400
Liver disease				
MASLD, n (%)	3488 (45%)	1261 (29.5%)	2227 (64%)	<.001
MASLD with significant fibrosis, n (%)	499 (6.4%)	71 (1.7%)	428 (12.3%)	<.001
MetALD, n (%)	542 (7%)	198 (4.6%)	344 (9.9%)	<.001
ALD, n (%)	175 (2.3%)	51 (1.2%)	124 (3.6%)	<.001
SLD total, n (%)	4434 (57.1%)	1649 (38.5%)	2785 (80.1%)	<.001
HCV (%)	178 (2.3%)	75 (1.7%)	103 (3%)	<.001
HBV (%)	90 (1.2%)	40 (0.9%)	50 (1.4%)	.043
Reported history of any liver disease (%)	364 (4.7%)	133 (3.1%)	231 (6.6%)	<.001
Reported history of liver fibrosis or cirrhosis (%)	24 (0.3%)	8 (0.2%)	16 (0.5%)	.039
Noninvasive tests				
FIB-4: median (IQR)	0.9 (0.6–1.3)	0.8 (0.5–1.3)	1.0 (0.6–1.4)	<.001
LSM, XL probe, n (%)	2061 (56.5%)	445 (21.6%)	1616 (78.4%)	<.001
LSM (kPa): median (IQR)	5 (4.1–6.2)	4.6 (3.9–5.6)	5.5 (4.5–7)	<.001
LSM ≥ 8kPa (%)	781 (10.1%)	171 (4%)	610 (17.5%)	<.001
CAP (dB/m): median (IQR)	260 (216–306)	232 (200–271)	297 (258–338)	<.001

Metabolic risk Population Defined as Individuals With Type 2 Diabetes, Abdominal Obesity With ≥1 Additional Metabolic Risk factor(s), or Elevated Liver Enzymes.^1^ Values Are Presented as Median (IQR) or Number (% Total). *P* Values Were Calculated Using the Wilcoxon or Fisher's Exact Test.

ALD, alcohol-related liver disease; BMI, body mass index; CAP, controlled attenuation parameter; EASL, European Association for the Study of the Liver; FIB-4, Fibrosis-4 Index; HBV, hepatitis B virus infection; HCV, hepatitis C virus infection; IQR, interquartile range; MASLD, metabolic associated steatotic liver disease; MetALD, metabolic dysfunction and alcohol-related liver disease; NHANES, National Health and Nutrition Examination Survey; SLD, steatotic liver disease; T2D, type 2 diabetes; TE, transient elastography.

The study population had a median age of 50 years (interquartile range 33–63) with balanced gender distribution (50% male). Among participants, 3479 (44.8%) met the metabolic risk population criteria, showing higher prevalence of key comorbidities compared to those not meeting criteria: arterial hypertension (53.4% vs 22.5%), T2D (31.1% vs 0%), and increased BMI (33.2 vs 25.5 kg/m^2^, all *P* < .001). Detailed baseline characteristics are presented in Table 1.

### Diagnostic Performance of the Screening Strategy

**Figure 2:**
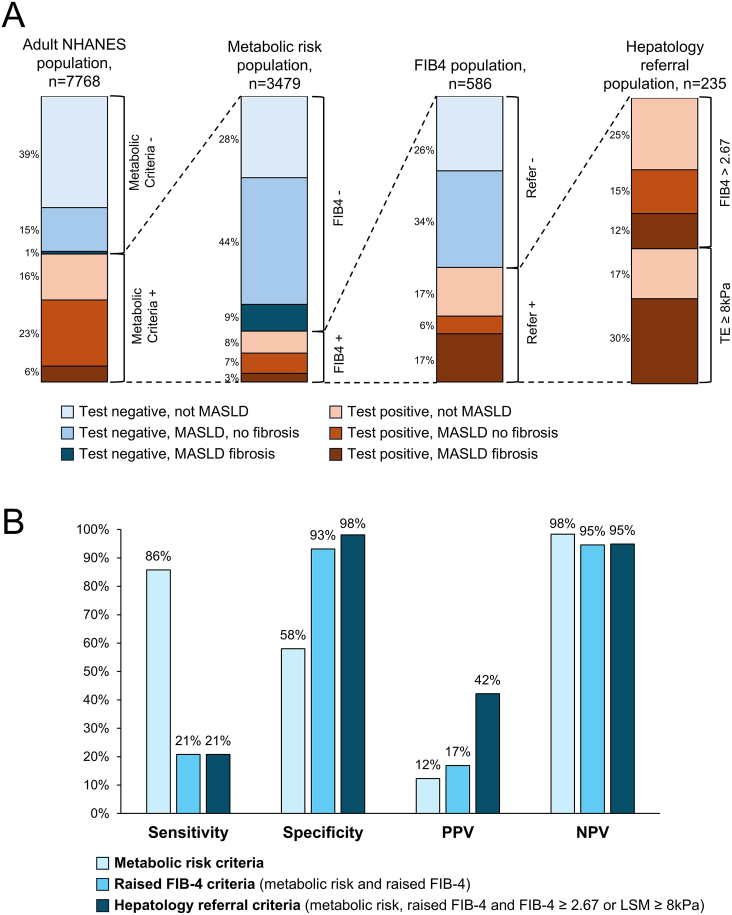
Stepwise application and diagnostic performance of the *multistep screening strategy* in NHANES 2017–2020. (A) Stepwise application of the 2024 multistep screening criteria. Stacked bar plots show the progressive stratification of the NHANES participants (n = 7768) through the screening algorithm. The population is divided into metabolic risk criteria (n = 3479), followed by raised FIB-4 criteria (n = 586) and hepatology referral criteria (n = 235). Bars illustrate the proportions of individuals testing negative or positive for MASLD without fibrosis, MASLD with significant fibrosis, or non-MASLD. (B) Diagnostic performance of the *multistep screening strategy*. Sensitivity, specificity, positive predictive value (PPV), and negative predictive value (NPV) are shown for the metabolic risk population (light blue), raised FIB-4 criteria (medium blue), and the overall multistep screening strategy meeting the hepatology referral criteria (dark blue). FIB-4, Fibrosis-4 Index; MASLD, metabolic dysfunction-associated steatotic liver disease; NPV, negative predictive value; PPV, positive predictive value; TE, transient elastography.

Sequential application of the multistep screening criteria revealed a stepwise reduction in the identified population (Figure 2A). Of the total cohort, 44.8% (n = 3479) met metabolic risk criteria, 7.5% (n = 586) met raised FIB-4 criteria, and 3.0% (n = 235) qualified for hepatology referral. The overall screening strategy demonstrated high specificity (98%, 95% CI 98%-98%) but limited sensitivity (21%, 95% CI 17%–25%) for detecting MASLD with significant fibrosis. The sensitivity and specificity for identifying significant fibrosis in MetALD was similar: 21% (95%, CI 13%–33%) and 97% (95%, CI 97%-97%) respectively (Table A2).

Analysis of the sequential screening steps showed sensitivity decreased markedly from 86% at the metabolic risk stage to 21% after FIB-4 assessment, while specificity increased from 58% to 93% (Figure 2B). Among patients meeting hepatology referral criteria (n = 235), 53% were identified through FIB-4 >2.67 and 47% through intermediate FIB-4 with LSM ≥8 kPa.

Within the hepatology referral population, a total of 171 (73%) patients had significant fibrosis; among them 99 had MASLD, 20 MetALD or alcohol-related liver disease and 40 had viral hepatitis. Sixty-four (27%) patients had no liver fibrosis (LSM< 8kPa) including 36 with MASLD, 4 with MetALD/alcohol-related liver disease and 7 with viral hepatitis.

### Subgroup Analyses

**Figure 3:**
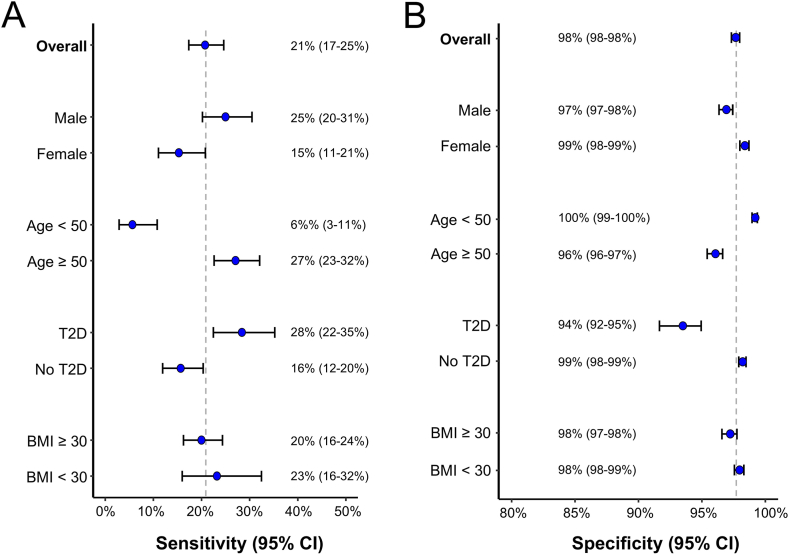
Subgroup analysis of the diagnostic performance of the *multistep screening strategy* by demographic and metabolic factors. (A) Sensitivity by subgroup. Forest plot showing the sensitivity (blue dots) with 95% confidence intervals (horizontal lines) for different subgroups, including sex, age, BMI, and T2D status. (B) Specificity by subgroup. Forest plot showing the specificity (blue dots) with 95% confidence intervals for the same subgroups. BMI, body mass index; CI, confidence interval; EASL, European Association for the Study of the Liver; T2D, type 2 diabetes.

The screening strategy's performance varied across demographic subgroups (Figure 3). Sensitivity was notably low among individuals aged <50 years (6%, 95% CI 3%–11%), women (15%, 95% CI 11%–21%) and patients without T2D (16%, 95% CI 12%–20%). Specificity was high across all subgroups, reaching 100% among those <50 years.

### Sensitivity Analysis

We conducted multiple sensitivity analyses to evaluate the robustness of our findings. First, we examined the impact of FIB-4 thresholds on screening performance. Replacing the age-adjusted FIB-4 thresholds with a uniform threshold of 1.3 slightly improved the overall strategy's sensitivity to 33% (95%, CI 29%–37%) while maintaining high specificity (98%, 95%, CI 97%–98%) (Figure A1).

Varying the definition of at-risk MASLD by increasing the LSM threshold to ≥12 kPa modestly improved sensitivity to 29% (95% CI 23%–37%), peaking at 35% (95% CI 27%–46%) with an LSM threshold of ≥15 kPa, while specificity remained high (97%–98%) (Figures A2 and A3). To address the limitations of TE-LSM as both a screening component and reference, we assessed the pathway's performance using alternative composite scores for at-risk MASLD. Sensitivity was 35% (95% CI 29%–41%) for Agile 3+ ≥0.68 (≥F3 fibrosis), 66% (95% CI 49%–79%) for Agile 4 ≥ 0.57 (cirrhosis), and 54% (95% CI 42%–66%) for FAST score ≥0.67 (high-risk MASH), all with high specificity (97%–98%) (Table A3). These analyses indicate that while sensitivity improves for more advanced disease definitions, it remains suboptimal for MASLD with significant fibrosis defined by LSM ≥8kPa.

### Modelling Performance of an Alternative Test to FIB-4

**Figure 4:**
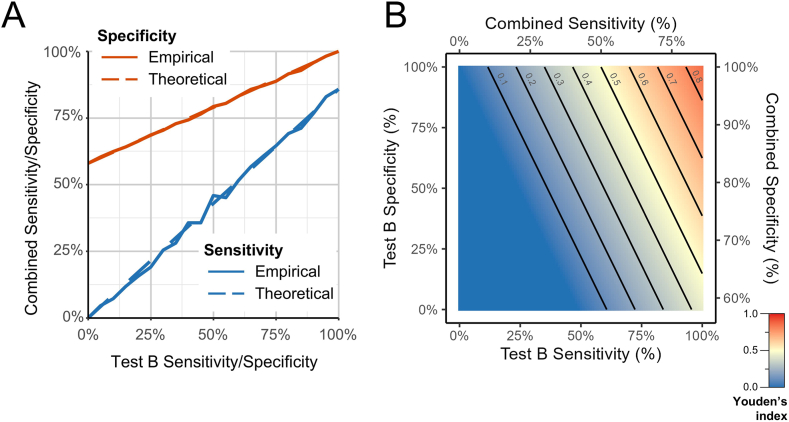
Modelling the performance of an alternative test (“test B”) to FIB-4 for identifying MASLD with significant fibrosis in patients with metabolic risk criteria. (A) Empirical (solid lines) and theoretical (dashed lines) models show the impact of varying test B sensitivity (blue) and specificity (orange) on combined diagnostic performance (ie, sequential metabolic risk criteria then test B). (B) Impact on overall screening performance. Heatmap showing the relationship between test B sensitivity, specificity, and combined screening performance, measured using the Youden Index. Contour lines represent Youden Index thresholds, with values increasing as test sensitivity and specificity improve. FIB-4, Fibrosis-4 Index; MASLD, metabolic dysfunction-associated steatotic liver disease; TE, transient elastography.

Given the current strategy's low sensitivity due to the FIB-4 step, we modeled replacing FIB-4 with a theoretical "test B" following the initial metabolic risk criteria. The pathway's combined sensitivity increased linearly with test B's sensitivity, capped at 86% (the sensitivity of metabolic risk criteria alone), while combined specificity improved from 58% to 100% with increasing test B specificity (Figure 4A). Using the Youden Index to evaluate overall performance (Figure 4B), we found that improving test B's sensitivity had a greater impact than improving its specificity. To achieve clinically meaningful targets (overall sensitivity ≥50%, specificity ≥90%), test B would require minimum sensitivity and specificity of 58% and 76%, respectively. This provides quantitative targets for developing better noninvasive screening tools for MASLD or MetALD with significant fibrosis.

## Discussion

This study evaluated the performance of a multistep screening strategy, reflective of current AASLD and EASL guidelines, for identifying individuals with MASLD or MetALD and significant fibrosis using a large, nationally representative US population from the NHANES cohort. Our primary finding is that while this strategy, which is based on the recent EASL guidelines, showed excellent specificity (98%), its sensitivity for identifying MASLD or MetALD with significant fibrosis (LSM ≥8 kPa) was critically low (21%). This poor sensitivity, particularly evident in younger individuals (6% sensitivity in those under 50 years) and those without T2D (16% sensitivity), raises concerns about the ability of this and similar screening strategies to identify at-risk patients for timely referral and intervention.

A major contributor to this low overall sensitivity is the reliance on the FIB-4 score with age-adjusted thresholds. Our finding of a 21% sensitivity (or 33% sensitivity with the use of a uniform FIB-4 threshold of 1.3) is in keeping with previous research, which has also shown limited sensitivity of FIB-4 for identifying significant fibrosis in general population settings.^8^^,^^13^^,^^18^ For instance, Graupera et al. found a FIB-4 sensitivity of 37% (threshold 1.3) in a general population,^18^ and De Vincentis et al. reported 27.7% for significant fibrosis, noting that age adjustment further compromised sensitivity.^8^ Our comprehensive sensitivity analyses reinforce these concerns; while the pathway's sensitivity improved modestly when targeting more advanced disease stages using higher LSM thresholds (eg, 29% for LSM ≥12 kPa) or composite scores like Agile 3+ (35% for ≥ F3 fibrosis), Agile 4 (66% for cirrhosis), and FAST (54% for high-risk MASH), it remained suboptimal for the primary target of detecting significant fibrosis (LSM ≥8kPa). These consistent findings across multiple large cohorts, and now including MetALD populations and alternative fibrosis definitions, underscore a fundamental limitation in the current screening paradigm in low-prevalence settings.

A critical consideration in pathway design is that while sequential testing typically increases specificity, it inherently reduces overall sensitivity with each additional step – a fundamental principle demonstrated in our analysis where sensitivity dropped from 86% after metabolic risk criteria to 21% after FIB-4 assessment. This trade-off must be carefully balanced when designing screening pathways, particularly in low-prevalence settings where maintaining adequate sensitivity is crucial.^19^ Our theoretical modelling provides insights for improving for improvement: to achieve an overall pathway sensitivity of ≥50% while maintaining specificity ≥90%, an alternative test replacing FIB-4 would require a minimum sensitivity of 58% and specificity of 76% when applied to those meeting metabolic risk criteria.

Routinely available indirect serum markers of liver fibrosis, such as the NAFLD Fibrosis Score, have shown similar or worse performance than FIB-4 in this context.^13^ The ELF test has shown a sensitivity of 67% to identify significant fibrosis in NAFLD in one systematic review.^20^ However, a recent prospective European study showed more modest performance, with a sensitivity of 34%–45% using a threshold of 9.8 to identify LSM ≥8kPa.^21^ While ELF has demonstrated prognostic value for hepatic decompensation and mortality,^22^ its widespread implementation remains limited by cost and availability. Imaging-based techniques for LSM, including TE, shear-wave elastography, and magnetic resonance elastography, are well-validated for identifying significant fibrosis.^1^^,^^6^^,^^23^^,^^24^ However, these strategies have limited availability, are costly and their implementation in primary care settings faces practical challenges. These limitations highlight the urgent need for novel, cost-effective screening tools that can be readily deployed in primary care. Our study distinctively validates the entire multistep screening pathway as recommended by major societies, including the initial metabolic risk assessment and subsequent noninvasive tests, in a general population, rather than focusing on individual test performance in isolation, which differentiates it from studies like the one by De Vincentis et al. that primarily assessed FIB-4 performance.^8^

This study has notable strengths, including the use of a large, nationally representative dataset, which enhances the generalizability of the findings to the broader US population. However, there are limitations that warrant consideration. Given the cross-sectional nature of NHANES we were not able to assess possible associations of FIB-4 with clinical outcomes. While previous studies have shown associations between FIB-4 and liver-related outcomes in MASLD cohorts,^25^^,^^26^ these findings primarily emerged from high-risk populations with frequent events, potentially limiting their applicability to general population screening with lower risk patients.^27^ Finally, to avoid circularity by using TE both as a component of the screening protocol and the reference standard we performed extensive sensitivity analyses using alternative, independently validated composite scores (FAST, Agile 3+, Agile 4). Although these scores also incorporate TE parameters, they represent distinct algorithms for identifying different strata of at-risk MASH or advanced fibrosis, thereby providing a more robust evaluation of the overall pathway's performance against various clinically relevant endpoints and strengthening the validity of our findings despite the absence of biopsy.

In conclusion, while the multistep screening strategy offers robust specificity, its limited sensitivity significantly hampers overall performance. This is especially problematic for detecting early to significant fibrosis (LSM ≥8kPa)—the primary intervention target of current guidelines—where the pathway performs poorly. Although sensitivity improves for more advanced fibrosis or high-risk MASH with alternative scores, the substantial risk of underdiagnosing significant fibrosis, particularly in younger individuals and those without T2D, persists. Clinicians should therefore maintain a low threshold for additional fibrosis assessment in patients with metabolic risk factors, especially if FIB-4 results are below current referral thresholds, to prevent missed early management opportunities.

## Footnotes

Material associated with this article can be found, in the online version, at doi:10.1016/j.gastha.2025.100747.
