# Supplementary material for: Validation of the American Association for the Study of Liver Disease/European Association for the Study of the Liver Multistep Screening Strategies for Metabolic Dysfunction-associated Steatotic Liver disease
Source: Gastro Hep Adv. 2025 Jul 10;4(10):100747. doi: 10.1016/j.gastha.2025.100747 (PMC12419083; doi:10.1016/j.gastha.2025.100747)
Supplement: Supplementary Material [file mmc1.docx]

**Supplementary Material**

**Validation of a Multi-Step Screening Strategy for MASLD with Significant Fibrosis**

**Authors**: Clémence Canivet^1^, Marie Ongaro^1^, Nicolas Conquet^1^, Laurent Spahr^1^, Nicolas Goossens^1, 2^

**Affiliations:**

^1^Division of Gastroenterology and Hepatology, Geneva University Hospitals, Geneva, Switzerland

^2^Division of Transplantation, Geneva University Hospitals, Geneva, Switzerland

## **Table of Contents**

[Supplementary Table 1 2](#_Toc186840112)

[Supplementary Figure 1 3](#_Toc186840113)

[Supplementary Figure 2 4](#_Toc186840114)

[Supplementary Figure 3 5](#_Toc186840115)

## **Supplementary Table 1**

Detailed reasons for ineligible or unavailable transient elastography (TE) data in the NHANES 2017-20 cohort.

| **Reason for exclusion of TE measurement** | | **n (%)** | |
| --- | --- | --- | --- |
| **Partial exam** | | **616 (32%)** | |
| - Fasting < 3 hours | | 266 | |
| - IQR / median > 30% | | 184 | |
| - < 10 valid measures | | 166 | |
| **Ineligible** | | **348 (18%)** | |
| - Pregnant/ Unable to get urine to test for pregnancy | | 148 | |
| - Other | | 200 | |
| **Not done** | | **961 (50%)** | |
| - Participant refusal | | 55 | |
| - Limited time | | 70 | |
| - Other | | 836 | |
| **Total** | | **1925 (100%)** | |
|  |  | |  |

## **Supplementary Table 2**

EASL/AASLD multi-step screening performance for significant fibrosis (TE ≥ 8kPa) in MASLD, MetALD, and combined subgroups. Table details prevalence, fibrosis proportion, and key diagnostic metrics (95% CIs).

| **Subgroup** | **MASLD** | **MetALD** | **MASLD or MetALD** |
| --- | --- | --- | --- |
| **Prevalence in cohort** | 45% | 7% | 52% |
| Proportion with TE ≥ 8kPa | 14% | 11% | 14% |
| **Sensitivity (95% CI)** | 21% (17-25%) | 21% (13-33%) | 21% (18-24%) |
| **Specificity (95% CI)** | 98% (98-98%) | 97% (97-97%) | 98% (98-99%) |
| **PPV (95% CI)** | 42% (36-49%) | 6% (3-9%) | 48% (41-54%) |
| **NPV (95%CI)** | 95% (94-95%) | 99% (99-99%) | 94% (94-95%) |

Abbreviations: AASLD, American Association for the Study of Liver Diseases; CI, Confidence Interval; EASL, European Association for the Study of the Liver; MASLD, Metabolic Dysfunction-Associated Steatotic Liver Disease; MetALD, Metabolic Dysfunction-Associated Alcohol-Related Liver Disease; NPV, Negative Predictive Value; PPV, Positive Predictive Value; TE, Transient Elastography.

## **Supplementary Table 3**

Sensitivity analysis of the AASLD/EASL multi-step MASLD screening pathway's diagnostic performance using varying definitions for the at-risk target population. This includes different transient elastography (TE) thresholds (TE ≥8kPa for significant fibrosis, TE ≥12kPa for advanced fibrosis) and established composite scores: AGILE 3+ (≥0.68 for advanced fibrosis ≥F3), AGILE 4 (≥0.57 for cirrhosis F4), and FAST (≥0.67 for high-risk MASH). The table shows the prevalence of each at-risk condition and the corresponding diagnostic metrics (sensitivity, specificity, PPV, NPV) with 95% CIs.

| **Subgroup** | **MASLD & TE ≥ 8kPa** | **MASLD & TE ≥ 12kPa** | **MASLD & AGILE 3+ ≥ 0.68** | **MASLD & AGILE 4 ≥ 0.57** | **MASLD & FAST ≥ 0.67** |
| --- | --- | --- | --- | --- | --- |
| **Prevalence in cohort** | 6.4% | 2.2% | 3.0% | 0.5% | 0.8% |
| **Sensitivity (95% CI)** | 21% (17-25%) | 29% (23-37%) | 35% (29-41%) | 66% (49-79%) | 54% (42-66%) |
| **Specificity (95% CI)** | 98% (98-98%) | 97% (97-98%) | 98% (97-98%) | 97% (97-97%) | 97% (97-98%) |
| **PPV (95% CI)** | 42% (36-49%) | 20% (15-26%) | 33% (27-39%) | 10% (7-14%) | 14% (10-19%) |
| **NPV (95%CI)** | 95% (94-95%) | 98% (98-99%) | 98% (98-98%) | 99.8% (99.7-99.9%) | 99.6% (99.4-99.7%) |

Abbreviations: AGILE (score based on AST, GGT, Platelets, LSM, Diabetes, Gender, and Age); CI, Confidence Interval; FAST, FibroScan-AST score; MASLD, Metabolic Dysfunction-Associated Steatotic Liver Disease; MASH, Metabolic Dysfunction-Associated Steatohepatitis; NPV, Negative Predictive Value; PPV, Positive Predictive Value; TE, Transient Elastography.

## **Supplementary Figure 1**

Sensitivity analysis of the *AASLD/EASL* *multi-step screening strategy* assessing the impact of changing the definition of raised FIB-4 from age-related thresholds to uniform FIB-4 > 1.3 to identify significant fibrosis in patients with MASLD.


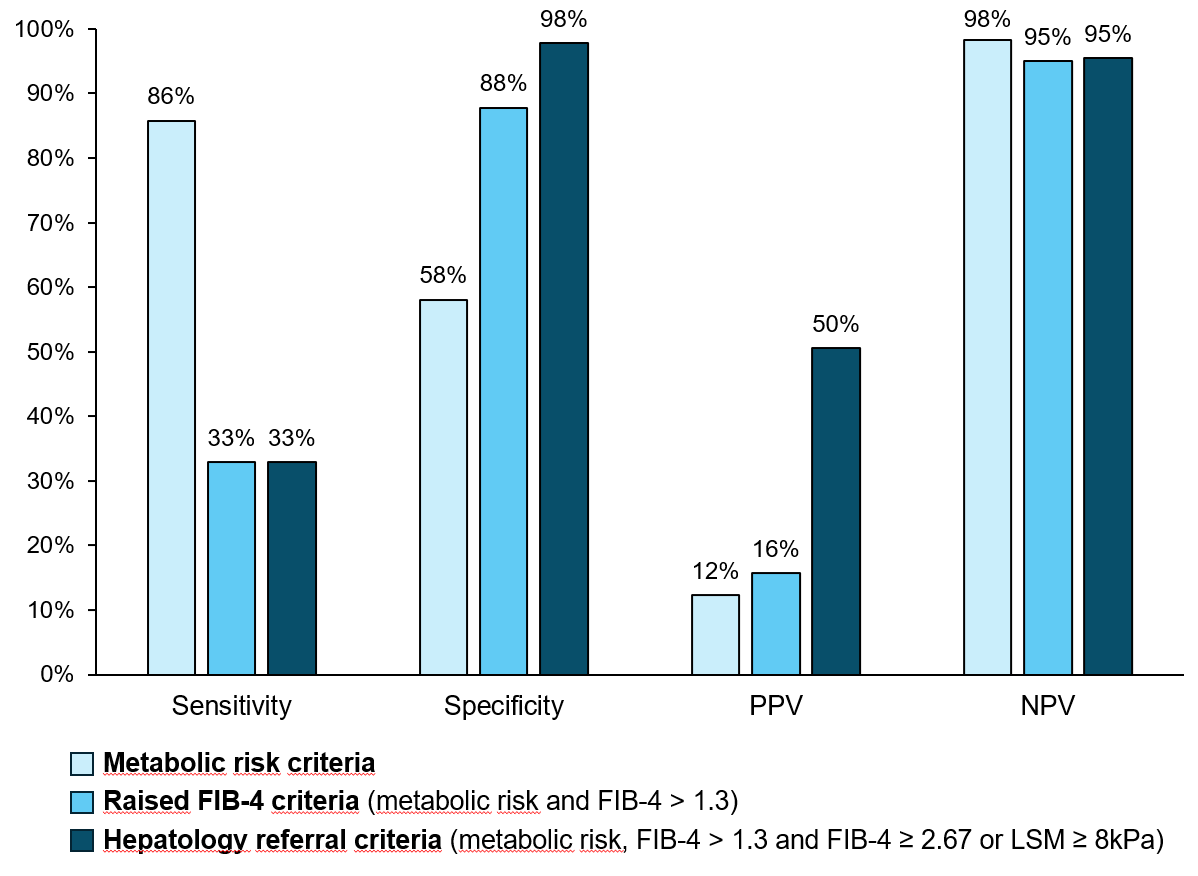


Abbreviations : AASLD, American Association for the Study of Liver Diseases; CI, Confidence Interval; EASL, European Association for the Study of the Liver; MASLD, Metabolic Dysfunction-Associated Steatotic Liver Disease; NPV, negative predictive value; PPV, positive predictive value.

## **Supplementary Figure 2**

Diagnostic performance of the *AASLD/EASL* *multi-step screening strategy* for identifying patients with MASLD and advanced fibrosis (MASLD and LSM ≥ 12 kPa) in NHANES 2017-20.


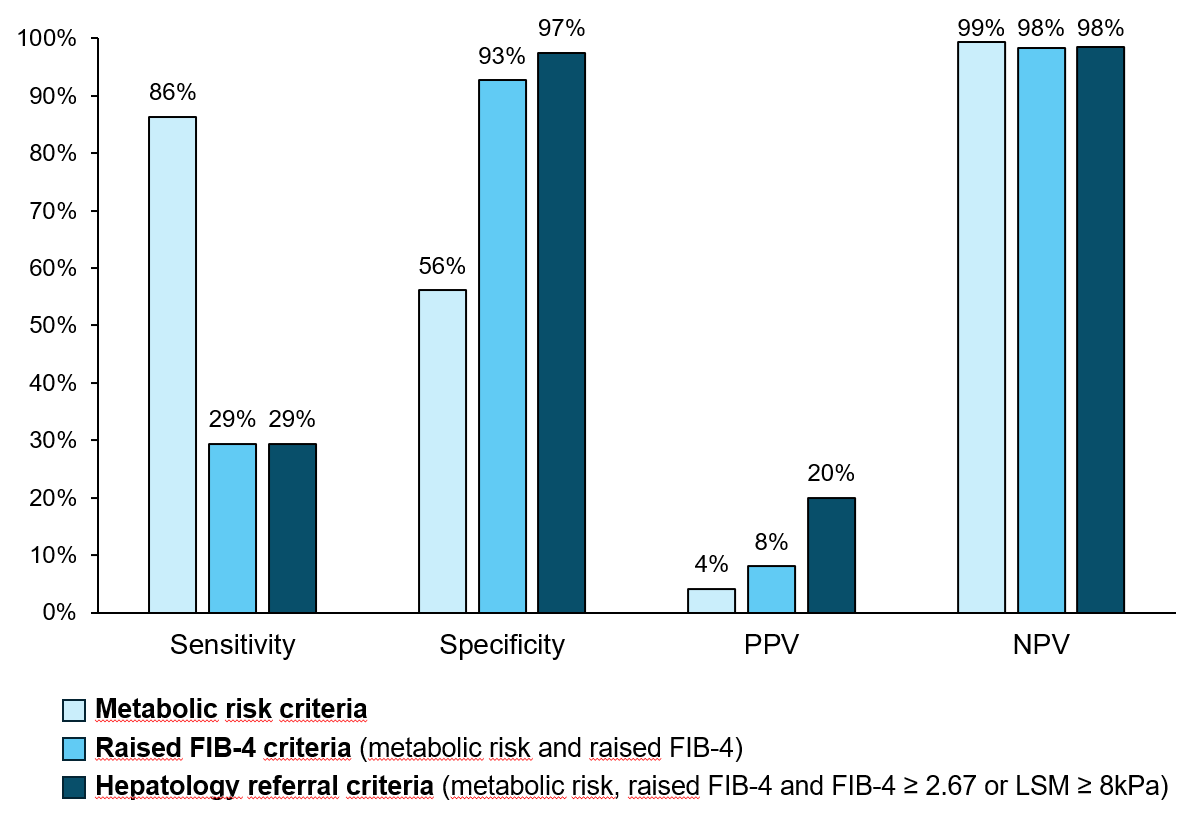


Abbreviations : AASLD, American Association for the Study of Liver Diseases; CI, Confidence Interval; EASL, European Association for the Study of the Liver; MASLD, Metabolic Dysfunction-Associated Steatotic Liver Disease; NPV, negative predictive value; PPV, positive predictive value.

## **Supplementary Figure 3**

Sensitivity analysis of the definition of MASLD with fibrosis and the performance of the *AASLD/EASL multi-step MASLD screening strategy*.

The figure evaluates the performance of the *multi-step MASLD screening strategy* using varying thresholds of LSM to define MASLD with fibrosis. While MASLD with fibrosis was originally defined as MASLD with LSM ≥8 kPa, this analysis assesses the algorithm’s sensitivity (blue) and specificity (orange) across alternative LSM thresholds. Shaded areas represent 95% confidence intervals, and dashed lines indicate best-fit curves. Vertical dotted lines highlight the standard thresholds of 8 kPa and 12 kPa.


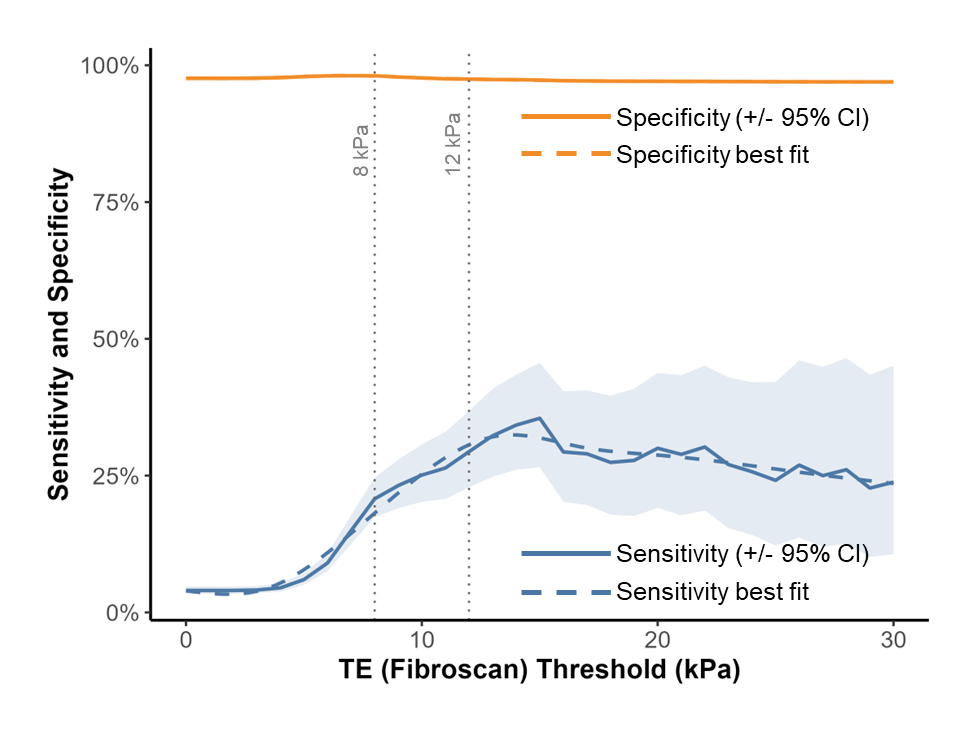


Abbreviations : CI, confidence interval; AASLD, American Association for the Study of Liver Diseases; CI, Confidence Interval; EASL, European Association for the Study of the Liver; LSM, liver stiffness measurement; MASLD, Metabolic Dysfunction-Associated Steatotic Liver Disease; TE, transient elastography
